# Awareness and Knowledge of Medical Interns in the Diagnosis and Management of Anaphylaxis in Medina Region, Saudi Arabia

**DOI:** 10.7759/cureus.67369

**Published:** 2024-08-21

**Authors:** Osama O Algrigri, Ibrahim S Alhejaili, Abdulmalik A Abogamel, Jawad W Alharbi, Mohammed A Kharabah, Abdulaziz F Alhejaili, Osama M Alharbi, Turki I Alharbi

**Affiliations:** 1 Pediatric Medicine, Taibah University, Medina, SAU; 2 College of Medicine, Taibah University, Medina, SAU

**Keywords:** medina, saudi arabia, immunological reaction, hypersensitivity, anaphylaxis, awareness

## Abstract

Background

Anaphylaxis is a severe hypersensitivity reaction caused by IgE-mediated mechanisms. This life-threatening condition affects multiple body systems. Global lifetime prevalence estimates for anaphylaxis range from 0.3% to 5.1%, with increasing incidence noted, particularly among young individuals. Symptoms range from mild skin manifestations like urticaria and pruritus to severe respiratory distress and hypotension.

Objectives

This study aimed to assess the knowledge level of medical interns in the Medina region, Saudi Arabia, regarding the diagnosis and management of anaphylaxis.

Method

A prospective cross-sectional study was conducted in hospitals in Saudi Arabia by delivering a questionnaire designed to assess the level of awareness and knowledge (including signs, symptoms, and management of anaphylaxis) among medical interns in the Medina region. The data collected were coded and analyzed using IBM SPSS Statistics for Windows, Version 22.0 (released 2013, IBM Corp., Armonk, NY).

Results

The study involved 291 interns from the universities in the Medina region. Most participants (275, 94.5%) correctly defined anaphylaxis and identified food allergies or insect stings as the main triggers (254, 87.3%). The participants demonstrated good knowledge of health education practices and anaphylaxis symptoms, such as the importance of carrying an adrenaline auto-injector (269, 92.4%) and recognizing syncope as a cardiovascular symptom (196, 67.4%). Regarding management, most correctly identified the initial step as removing the allergen (226, 77.7%) and epinephrine as the preferred medication (256, 88.0%). Significant gender differences were observed in the knowledge of management aspects (P = 0.001, P = 0.002, P = 0.049, P = 0.004, P = 0.001).

Conclusion

The study found that most participants had a good understanding of the definition of anaphylaxis and its most common triggers. The participants also demonstrated knowledge of symptoms and signs associated with anaphylaxis and the appropriate management of anaphylaxis. However, there were some differences in knowledge between females and males, suggesting that further education and awareness campaigns may be needed to ensure an accurate and consistent understanding of anaphylaxis among both genders. Overall, the study highlights the importance of education and awareness in effectively managing anaphylaxis and preventing its complications.

## Introduction

Anaphylaxis is a severe hypersensitivity reaction caused by IgE-mediated reaction [1]. It can also happen due to IgG or immune complex complement-related immunological reactions that lead to the degranulation of mast cells [2]. It is a life-threatening reaction affecting multiple systems in the body. The lifetime prevalence estimates for anaphylaxis range from 0.3% to 5.1% and appear to be increasing globally, particularly in young people. Symptoms of anaphylaxis vary from non-severe skin problems such as urticaria and pruritus to severe conditions such as respiratory distress and hypotension. Causes of anaphylaxis vary from country to country [3]. Foods are the most common triggers for anaphylactic reactions, followed by drugs, insect stings, and idiopathic anaphylaxis (anaphylaxis of unknown cause) [4].

The incidence and prevalence of anaphylaxis have increased in the last two decades. The overall global incidence of anaphylaxis is reported to be between 50 and 112 episodes per 100,000 person-years [2,3], and the lifetime prevalence estimates range from 0.3% to 5.1%. Data show that more reported cases of anaphylaxis occurred in those younger than 18 years of age [3,4]. Anaphylaxis mortality rate also varies from country to country; in the USA, it was reported to be between 0.63 and 0.73 per million persons [5].

The drug of choice for treating anaphylaxis is epinephrine. Patients and caregivers should be educated on the use of epinephrine and emergency management of anaphylaxis. Delays in epinephrine administration have been associated with increased fatality [2,3]. In this study, we aimed to conduct survey-based research about the knowledge of medical interns regarding the diagnosis and management of anaphylaxis in the Medina region and assess if they require more education and training.

## Materials and methods

This prospective cross-sectional questionnaire-based study (see Appendix) was conducted to assess medical interns practicing in various specialties within the Medina region, Saudi Arabia. Invitations were sent electronically to eligible participants, targeting all practicing medical interns in Medina’s primary, secondary, and tertiary healthcare facilities and hospitals. Taibah University, College of Medicine Research Ethics Committee (CM - REC) issued approval STU-22-20.

The inclusion criteria involved all practicing medical interns within the specified region, resulting in a total sample size of 291 medical interns, with 194 from Taibah University and 97 from Al Rayyan Medical College. The sample size was calculated using the Cochrane formula: n = Z²p (1 − p)/d², where n represents the sample size, Z is the critical value for a 95% confidence level, p is the assumed proportion (set at 50%), and d is the margin of error (6%). The initial calculation indicated a minimum required sample size of 267. To enhance reliability, however, we increased the sample to 291 complete medical records.

Data collection was executed through a structured questionnaire distributed to the participants. The pilot study participants were excluded from the main study. The reliability of the questionnaire was assessed using Cronbach's alpha, yielding a value of 0.842, showing a good level of internal consistency. Following the incorporation of adjustments prompted by their feedback, the questionnaire was adjusted accordingly. The duration of the study spanned 12 months between the period of July 2023 and July 2023, following the receipt of ethical approval.

Participants' knowledge regarding anaphylaxis etiology, pathophysiology, symptoms, and management was categorized into four levels: "good," "average," "less than average," and "lack of knowledge." This categorization was based on their scores from a validated knowledge assessment questionnaire, with predefined cut-off points determining each level. Scores were assigned to each response, and participants were categorized according to their total score. Statistical analysis involved using the medical intern’s t-test to ascertain the significance of differences between the mean values of two continuous variables.

Ethical considerations were carefully observed throughout the study. The participants were informed about the study's procedures and purpose, and their informed consent was obtained. High confidentiality was assured regarding the identity and information of the participants, aligning with ethical standards for research involving human subjects.

## Results

Table 1 shows that the majority of the participants (275, 94.5%) knew the definition of anaphylaxis as a potentially life-threatening type 1 hypersensitivity reaction. On the other hand, in regard to the most common trigger of anaphylaxis among young age, most of the participants (254, 87.3%) identified food allergies or insect stings as the main trigger of anaphylaxis among the young age group.

**Table 1:** Prevalence of knowledge of etiology and pathophysiology (N = 291) Etiology and pathophysiology knowledge are presented in frequencies (n) and proportions (%).

Variables	Category	n (%)
What is the definition of anaphylaxis?	Several Immunological responses to infectious organisms	7 (2.4%)
	Several and potentially life-threatening type 1 hypersensitivity reaction	275 (94.5%)
	Skin rash caused by allergic reaction	4 (1.4%)
	Mild to moderate allergic reaction to external trigger	5 (1.7%)
The most common trigger of anaphylaxis among the young age group is?	Iatrogenic (drugs or radiocontrast media)	17 (5.8%)
	Exercise	9 (3.1%)
	Food allergies or insect stings	254 (87.3%
	Idiopathic	11 (3.8%)

Table 2 shows that most of the respondents (194, 66.7%) knew that an adrenaline administration should be carried out within minutes to hours after experiencing anaphylaxis. In terms of cardiovascular symptoms/signs of anaphylaxis, most of the respondents (196, 67.4%) were aware of syncope as a symptom. For gastrointestinal symptoms/signs of anaphylaxis, most of the respondents (239, 82.1%) recognized nausea/vomiting as a symptom. In addition, most of the participants (216, 74.2%) recognized pleuritic chest pain as not being one of the symptoms/signs of anaphylaxis. When it comes to neurological symptoms/signs of anaphylaxis, most of the respondents (230, 79.0%) identified confusion as a symptom. Furthermore, more than half of the participants (171, 58.8%) recognized vesicular rash as one of the skin changes that is not associated with anaphylaxis. Moreover, a majority of the participants (236, 90.3%) believed that a decreased level of consciousness indicates a severe reaction in anaphylaxis. In terms of diagnostic criteria for anaphylaxis, most of the respondents (239, 82.1%) did not recognize fever in a patient with a known previous attack of anaphylaxis as a form of diagnostic criteria for anaphylaxis.

**Table 2:** Prevalence of knowledge about signs and symptoms of anaphylaxis (N = 291) Knowledge of signs and symptoms of anaphylaxis is presented in frequencies (n) and proportions (%). The level of awareness among the interns is statistically significant at the P < 0.05* level.

Variables	Category	n (%)
Should a person who has experienced anaphylaxis carry an adrenaline auto-injector (administration) within them?	Seconds	96 (33.0%)
	Minutes to hours	194 (66.7%)
	It takes two to five days	1 (0.3%)
	Delayed onset takes more than five days	0 (0.0%)
Which one of the following is a cardiovascular symptom/sign of anaphylaxis?	Hypertension	9 (3.0%)
	Bradycardia	57 (19.6%)
	Syncope	196 (67.4%)
	Increased body temperature	29 (10.0%)
Which one of the following is a gastrointestinal symptom/sign of anaphylaxis?	Hematemesis	10 (3.5%)
	Constipation	34 (11.7%)
	Heartburn	8 (2.7%)
Which one of the following is not a symptom/sign of anaphylaxis?	Dyspnea	27 (9.3%)
	Wheezing	33 (11.3%)
	Hypoxia	15 (5.2%)
	Pleuritic chest pain	216 (74.2%)
Which one of the following could be a neurological symptom/sign of anaphylaxis?	Confusion	230 (79.0%)
	Lower-limb weakness	9 (3.1%)
	Double vision	11 (3.8%)
	Statisti tremors	41 (14.1%)
Which one of the following skin changes is not associated with anaphylaxis?	Erytherna	26 (8.9%)
	Swelling of the eyelids and lips	26 (8.9%)
	Urticarial rash	68 (23.4%)
	vesicular rash	171 (58.8%)
What is the symptom of anaphylaxis that indicates a severe reaction?	Skin erythema	6 (2.1%)
	Nasal congestion	2 (0.7%)
	Decreased level of consciousness	263 (90.3%)
	Tachycardia	20 (6.9%)
Which one of the following is not a form of the diagnostic criteria for anaphylaxis?	Involvement of two or more organ systems after exposure to likely allergen	30 (10.3%)
	Hypotension after exposure to known allergen for the patient	15 (5.2%)
	Fever in a patient with a known previous attack of anaphylaxis	239 (82.1%)
	Involvement of the respiratory or cardiovascular system with skin symptoms	7 (2.4%)

Table 3 shows that most of the participants (226, 77.7%) were aware that the first step in managing anaphylaxis is to remove the inciting allergen. In regard to the most effective medication for treating anaphylaxis, most of the participants (256, 88.0%) knew epinephrine as the most effective medication for treating anaphylaxis. In terms of the recommended dose of epinephrine, slightly more than half of the participants (151, 51.9%) were aware of 0.5 mg as the recommended dose for adults, while 170 (58.4%) of the participants were aware of 0.01 mg/kg as the recommended dose of epinephrine for children aged six months to six years. Most of the participants (232, 79.7%) were aware of intramuscular as the recommended route of administration for epinephrine in treating anaphylaxis. More so, 222 (76.3%) of the participants were aware of loop diuretics as one option that is not considered in the treatment of refractory anaphylaxis. In addition, most of the participants (247, 84.9%) recognized aspirin as a medicine that is not considered in adjunctive therapy for anaphylaxis. In addition, the majority (279, 95.1%) of the participants were aware that it is possible for a person to experience anaphylaxis without having a history of allergies. Furthermore, 269 (92.4%) of the participants were aware that a person who has experienced anaphylaxis should carry an adrenaline auto-injector with them. Nevertheless, most of the participants (260, 89.3%) were aware that it is necessary to stay in the hospital for observation after being treated for anaphylaxis. Finally, most of the participants (259, 89.0%) were aware that anaphylaxis can recur after the initial episode has been treated. Among the variables, there is a significant difference in the level of knowledge between males and females in regard to the first step in managing anaphylaxis (p = 0.001), the most effective medication for treating anaphylaxis (p = 0.001), the recommended dose of epinephrine for treating anaphylaxis in adults (p = 0.002), recommended route of administration for epinephrine (p = 0.049), adjunctive therapy for anaphylaxis (p = 0.004), and possibility of anaphylaxis recurrence (p < 0.001).

**Table 3:** Prevalence of knowledge about the management of anaphylaxis (N = 291) Anaphylaxis management knowledge is presented in frequencies (n) and proportions (%). The level of awareness among the females and males was statistically significant at the P < 0.05* level.

Variables	Total = 291 (%)	Female = 119 (%)	Male = 172 (%)	P-value*
What is the first step in managing anaphylaxis				*0.001
Remove inciting allergen	226 (77.7%)	106 (89.1%)	120 (69.8%)	
Take antihistamine	61 (21.0%)	12 (10.1%)	49 (28.5%)	
Drink plenty of water	1 (0.3%)	0 (0.0%)	1 (0.6%)	
Rest	3 (1.0%)	1 (0.8%)	2 (1.2%)	
What is the most effective medication for treating anaphylaxis?				*0.001
Aspirin	14 (4.8%)	8 (6.7%)	6 (3.5%)	
Antihistamine	18 (6.2%)	15 (12.6%)	3 (1.7%)	
Epinephrine	256 (88.0%)	94 (79.0%)	162 (94.2%)	
Corticosteroid	3 (1.0%)	2 (1.7%)	1 (0.6%)	
What is the recommended dose of epinephrine for treating anaphylaxis in adults?				*0.002
mg 0.1	56 (19.2%)	25 (21.0%)	31 (18.1%)	
0.2 mg	60 (20.6%)	12 (10.1%)	48 (27.9%)	
0.5 mg	151 (51.9%)	73 (61.3%)	78 (45.3%)	
1 mg	24 (8.2%)	9 (7.6%)	15 (8.7%)	
What is the recommended dose of epinephrine for treating anaphylaxis in children from six months to six years?				0.439
0.01 mg/kg	170 (58.4%)	75 (63.0%)	95 (55.2%)	
0.1 mg/kg	86 (29.6%)	30 (25.2%)	56 (32.6%)	
0.03 mg/kg	34 (11.7%)	14 (11.8%)	20 (11.6%)	
1 mg/kg	1 (0.3%)	0 (0.0%)	1 (0.6%)	
What is the recommended route of administration for adrenaline (epinephrine) for treating anaphylaxis?				*0.049
Oral	5 (1.7%)	1 (0.8%)	4 (2.3%)	
Intramuscular	232 (79.7%)	103 (86.6%)	129 (75%)	
Intravenous	46 (15.9%)	11 (9.2%)	35 (20.4%)	
Subcutaneous	8 (2.7%)	4 (3.4%)	4 (2.3%)	
Which of the following is not considered an option in the treatment of refractory anaphylaxis?				0.077
IV epinephrine infusion	25 (8.6%)	6 (5.0%)	19 (11.1%)	
IV glucagon	33 (11.3%)	19 (16.0%)	14 (8.1%)	
Loop diuretics	222 (76.3%)	90 (75.6%)	132 (76.7%)	
Vasopressor	11 (3.8%)	4 (3.4%)	7 (4.1%)	
Which of the following is not considered as adjunctive therapy for anaphylaxis?				*0.004
Aspirin	247 (84.9%)	102 (85.7%)	145 (84.3%)	
Antihistamines	13 (4.5%)	2 (1.7%)	11 (6.4%)	
Corticosteroids	21 (7.2%)	14 (11.8%)	7 (4.1%)	
Oxygen therapy	10 (3.4%)	1 (0.8%)	9 (5.2%)	
Is it possible for a person to experience anaphylaxis without having a history of allergies?				0.064
Yes	279 (95.1%)	111 (93.3%)	169 (98.3%)	
No	12 (4.1%)	8 (6.7%)	3 (1.7%)	
Should a person who has experienced anaphylaxis carry an adrenaline auto-Injector within them?				0.366
Yes	269 (92.4%)	108 (90.8%)	169 (92.4%)	
No	22 (7.6%)	11 (9.2%)	11 (7.6%)	
Is it necessary to stay in hospital for observation after being treated for anaphylaxis				0.369
Yes	260 (89.3%)	104 (87.4%)	156 (90.7%)	
No	31 (10.7%)	15 (12.6%)	16 (9.3%)	
Can anaphylaxis recur after initial episode has been treated				*0.001
Yes	259 (89.0%)	117 (98.3%)	142 (82.6%)	
No	32 (11.0%)	2 (1.7%)	30 (17.4%)	

**Figure 1:**
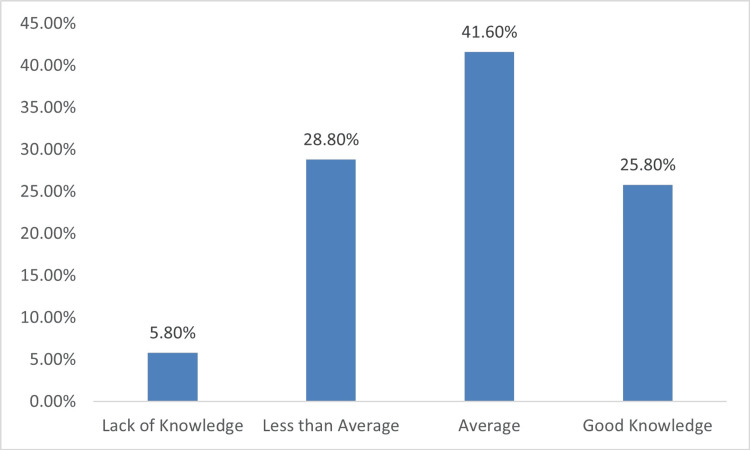
Level of knowledge about anaphylaxis (N = 291)

Figure 1 shows that 121 (41.6%) of the participants had an average level of knowledge regarding anaphylaxis etiology, pathophysiology, symptoms, and management, while 75 (25.8%) of the participants demonstrated to have a good level of knowledge. On the other hand, 78 (26.8%) participants had less than average level of knowledge, and only 17 (5.8%) were shown to lack knowledge about anaphylaxis etiology, pathophysiology, symptoms, and management.

## Discussion

The aim of this study was to assess the knowledge level regarding the diagnosis and management of anaphylaxis among medical interns in the Medina region. The finding of the study revealed that most of the participants (275, 94.5%) correctly defined anaphylaxis as a potentially life-threatening type 1 hypersensitivity reaction. This suggests that there is a good understanding of the definition of anaphylaxis among the study participants. Regarding the most common trigger of anaphylaxis among young age groups, 254 (87.3%) of the participants were knowledgeable of food allergies or insect stings as the main trigger, which suggests that there is a substantial knowledge of the anaphylaxis triggers. This finding concurs with the findings obtained in the study by Drupad and Nagabushan, which found that healthcare providers have a high level of knowledge about anaphylaxis and its management [6]. Most respondents, specifically 66.7%, believe that carrying an adrenaline auto-injector within minutes to hours after experiencing anaphylaxis is important.

Moving on to cardiovascular symptoms and signs of anaphylaxis, most respondents (67.4%) correctly identified syncope as a symptom. For gastrointestinal symptoms, 82.1% of the participants recognized nausea/vomiting as a symptom of anaphylaxis. When it comes to neurological symptoms, the majority (79.0%) correctly identified confusion as a symptom of anaphylaxis. In addition, 90.3% of the participants believed that a decreased level of consciousness indicates a severe reaction in anaphylaxis. In terms of symptoms and signs that are not associated with anaphylaxis, 194 (66.7%) participants correctly identified that pleuritic chest pain is not a symptom. Likewise, 171 (58.8%) participants recognized that vesicular rash skin changes are not associated with anaphylaxis. Regarding diagnostic criteria of anaphylaxis, most respondents (82.1%) did not consider fever in a patient with a known previous attack of anaphylaxis to be a diagnostic criterion. These findings contradict with the findings obtained in the study by Baççioğlu and Yilmazel, which found that the level of knowledge of anaphylaxis among healthcare providers was found to be low, highlighting the need for increased education and awareness in this area [7].

The findings of the study indicate that most participants have a good level of knowledge about anaphylaxis management. In terms of the first step in managing anaphylaxis, 77.7% of the participants correctly identified that the first step is to remove the inciting allergen. Furthermore, the study revealed that the respondents had substantial knowledge of anaphylaxis management, with 256 (88.0%) of them indicating epinephrine as the most effective medication for treating anaphylaxis. Moreover, the study revealed that a considerable number of the participants knew the recommended dose of epinephrine for treating anaphylaxis with more than half 151 (51.9%), indicating 0.5 mg as the recommended dose for treating anaphylaxis among the adults and 170 (58.4%) noting 0.01 mg/kg as the recommended dose of epinephrine for treating anaphylaxis among the children aged six months to six years. These findings are consistent with the findings obtained in the study by Urrutia et al., which assessed the knowledge of asthma, food allergies, and anaphylaxis among teachers, parents, and university students. In their study, they noted that more than half of the participants had substantial knowledge regarding asthma, food allergies, and anaphylaxis [8]. Moreover, the findings of this study indicated that most of the respondents (222, 76.3%) were aware that loop diuretics are not considered an option for treating refractory anaphylaxis. Generally, most of the participants were knowledgeable of various factors used in the management of anaphylaxis and those that are not used in the management. As the study reveals, 259 (89.0%) of the respondents were aware that anaphylaxis can recur after the initial episode has been treated. Among the variables, there is a significant difference in the level of knowledge between males and females in regard to the first step in managing anaphylaxis (p = 0.001), the most effective medication for treating anaphylaxis (p = 0.001), the recommended dose of epinephrine for treating anaphylaxis in adults (p = 0.002), the recommended route of administration for epinephrine (p = 0.049), adjunctive therapy for anaphylaxis (p = 0.004), and the possibility of anaphylaxis recurrence (p = 0.001).

Overall, the results of the study indicate that a significant number of participants had a good level of knowledge about anaphylaxis. Approximately 25.8% of the participants scored 18 or higher on the knowledge assessment, indicating a good understanding of anaphylaxis. On the other hand, a considerable proportion of participants had average knowledge about anaphylaxis. About 41.6% of the participants scored between 14 and 18 on the assessment, indicating an average level of knowledge. This finding aligns with findings obtained in the study by Patnaik et al., which investigated knowledge, attitude, and practice regarding anaphylaxis among pediatric healthcare providers, revealing challenges and areas for improvement in their understanding and management of anaphylactic reactions [9].

One limitation of the study is the lack of diversity in the participants, as all interns were from a specific geographical location, which may limit the generalizability of the results. In addition, the study only included interns who received and responded to the survey via email, potentially excluding those without access, leading to a convenience sample that may not represent all interns in the region. Moreover, no sample size calculation was performed, further affecting the representativeness of the findings. Finally, the questionnaire used in the study was not validated, which could have led to varying interpretations of the questions by participants, potentially influencing their responses.

## Conclusions

Our study found that most of the participants had an average understanding of the definition of anaphylaxis and its most common triggers. The participants also had good knowledge regarding symptoms and signs associated with anaphylaxis and its appropriate management strategies. However, there were some differences in knowledge between females and males, suggesting that further education and awareness campaigns may be needed to ensure an accurate and consistent understanding of anaphylaxis among both genders. Overall, the study highlights the importance of education and awareness in effectively managing anaphylaxis and preventing its severe complications.

## Appendices

**Table 4:** Questionnaire of the study

A. Etiology and pathophysiology (10%)
1. The definition of anaphylaxis is? a) Severe immunological response to infectious organisms. b) Severe and potentially life-threatening type 1 hypersensitivity reaction. C) Skin rashes caused by allergic reaction. d) Mild to moderate allergic reaction to external triggers.
2. The most common trigger of anaphylaxis among the young age group is? a) Iatrogenic (drugs or radiocontrast media. b) Exercise. c) Food allergies or insect stings. d) Idiopathic.
B. Signs and symptoms (40%)
3. How quickly do symptoms of anaphylaxis appear after exposure to a trigger? a) Seconds. b) Minutes to hours. c) It takes 2-5 days. d) Delayed onset takes more than 5 days.
4. Which one of the following is a cardiovascular symptom/sign of anaphylaxis? a) Hypertension. b) Bradycardia. c) Syncope. d) Increased body temperature.
5. Which one of the following is a gastrointestinal symptom/sign of anaphylaxis? a) Hematemesis. b) Nausea/vomiting. c) Constipation. d) Heartburn.
6. Which one of the following is not a respiratory symptom/sign of anaphylaxis? a) Dyspnea. b) Wheezing. c) Hypoxia. d) Pleuritic chest pain.
7. Which one of the following could be a neurological symptom/sign of anaphylaxis? a) Confusion. b) Lower limb weakness. c) Double vision. d) Static tremors.
8. Which of the following skin changes is not associated with anaphylaxis? a) Erythema. b) Swelling of the eyelids and lips. c) Urticaria. d) Vesicular rash.
9. What is the symptom of anaphylaxis that indicates a severe reaction? a) Skin erythema. b) Nasal congestion. c) Decreased level of consciousness. d) Tachycardia.
10. Which one of the following does not form the diagnostic criteria for anaphylaxis? a) Involvement of 2 or more organ systems after exposure to likely allergen. b) Hypotension after exposure to known allergens for the patient. c) Fever in a patient with a known previous attack of anaphylaxis. d) Involvement of the respiratory or cardiovascular system with skin symptoms.
C. Management (50%)
11. What is the first step in managing anaphylaxis? a) Remove inciting allergen. b) Take antihistamine. c) Drink plenty of water. d) Rest.
12. What is the most effective medication for treating anaphylaxis? a) Aspirin. b) Antihistamine. c) Epinephrine. d) Corticosteroid.
13. What is the recommended dose of epinephrine for treating anaphylaxis in adults? a) 0.1 mg. b) 0.2 mg. c) 0.5 mg. d) 1 mg.
13. What is the recommended dose of epinephrine for treating anaphylaxis in children from 6 months to 6 years? a) 0.1 mg. b) 0.2 mg. c) 0.3 mg. d) 0.5 mg.
14. What is the recommended route of administration for adrenaline (epinephrine) in treating anaphylaxis? a) Oral. b) Intramuscular. c) Intravenous. d) Subcutaneous.
15. Which one of the following is not considered an option in the treatment of refractory anaphylaxis? a) IV epinephrine infusion. b) IV glucagon. c) Loop diuretics. d) Vasopressors.
16. Which one of the following is not considered adjunctive therapy for anaphylaxis? a) Aspirin. b) Antihistamines. c) Corticosteroids. d) Oxygen therapy.
17. Can anaphylaxis recur after the initial episode has been treated? a) Yes. b) No.
18. Is it necessary to stay at a hospital for observation after being treated for anaphylaxis? a) Yes. b) No.
19. Should a person who has experienced anaphylaxis carry an adrenaline auto-injector with them? a) Yes. b) No.
20. Is it possible for a person to experience anaphylaxis without having a history of allergies? a) Yes. b) No.
Scoring system
Interpretation of the result: Below 10 points: lack of knowledge, 10-14 points: less than average, 14-18 points: average, 18+ points: good knowledge
Category number of questions (one point for each)
1. Etiology and pathophysiology: 1-2 questions
2. Symptoms and signs: 3-10 questions
3. Management: 11-20 questions
